# Supplementary material for: Characterization of Copper/Zinc Superoxide Dismutase Activity on Phascolosoma esculenta (Sipuncula: Phascolosomatidea) and Its Protection from Oxidative Stress Induced by Cadmium
Source: Int J Mol Sci. 2022 Oct 12;23(20):12136. doi: 10.3390/ijms232012136 (PMC9602484; doi:10.3390/ijms232012136)
Supplement: Supplementary file 1 [file ijms-23-12136-s001.zip › ijms-1960890-supplementary.pdf]

Supplementary Materials:

Table S1. The primers squences

Primer/probe name	Primer sequence (5'-3')	Purpose
Cu/Zn SOD-F	AACCCAGGAAACAAGGAACA	PCR
Cu/Zn SOD-R	TTGCCAGTGGTCTTGCTCA	PCR
3' -Cu/Zn SOD-F1	GGATTCCCAGATTCCATTG	3' RACE
3' -Cu/Zn SOD-F2	GAGCAAGACCACTGGCAAT	3' RACE
5' -Cu/Zn SOD-R1	AATGACTCCACAAGCCAGCCGA	5' RACE
5' -KIF3B-R2	GCATTGCCAGTGGTCTTGCTCA	5' RACE
GADPH-F	CCAGAACATCATCCCAGCA	qPCR
GADPH-R	ACGAACAGGGACACGGAAG	qPCR
Cu/Zn SOD-RT-F	TTCAACCCAGGAAACAAGG	qPCR
Cu/Zn SOD-RT-R	CACACAATGGAATCTGGGAA	qPCR

Table S2. Species and GenBank accession numbers of Cu/ZnSOD sequences used for multiple alignment and phylogenetic analysis

Species name	Gene name	Genbank number	Length
Homo sapiens	ic Cu/Zn SOD	NP_000445.1	154 aa
Rattus norvegicus	ic Cu/Zn SOD	NP_058746.1	154 aa
Mus musculus	ic Cu/Zn SOD	NP_035564.1	154 aa
Gallus gallus	ic Cu/Zn SOD	NP_990395.1	154 aa
Melopsittacus undulatus	ic Cu/Zn SOD	AAO72711.1	154 aa
Danio rerio	ic Cu/Zn SOD	CAA72925.1	154 aa
Salmo salar	ic Cu/Zn SOD	NP_001117059.1	154 aa
Apis mellifera	ic Cu/Zn SOD	NP_001171498.1	152 aa
Crassostrea gigas	ic Cu/Zn SOD	XP_011426932.1	156 aa
Pinctada fucata	ic Cu/Zn SOD	AFM75822.1	156 aa
Schistosoma japonicum	ic Cu/Zn SOD	AAW25513.1	153 aa
Caenorhabditis elegans	ic Cu/Zn SOD	NP_001021957.1	158 aa
Loa loa	ic Cu/Zn SOD	EFO15395.1	156 aa
Homo sapiens	ec Cu/Zn SOD	NP_003093.2	240 aa
Mus musculus	ec Cu/Zn SOD	NP_035565.1	251 aa
Oryctolagus cuniculus	ec Cu/Zn SOD	NP_001076101.1	244 aa
Salmo salar	ec Cu/Zn SOD	NP_001134234.1	215 aa
Danio rerio	ec Cu/Zn SOD	ACY78689.1	213 aa
Dastarcus helophoroides	ec Cu/Zn SOD	AIG92783.1	222 aa
Dictyocaulus viviparus	ec Cu/Zn SOD	ABS12246.1	186 aa
